# A Private Nurse's Earnings

**Published:** 1920-05-15

**Authors:** 


*To the Editor of* The Hospital.

### A Private Nurse's Earnings.

*To the Editor of* The Hospital.

Sir,—As a private nurse working on my own account I beg to challenge the statement in The Hospital of May 1 that "the income earned by a well trained private nurse compares more than favourably with many other spheres of women's work." The statement that a private nurse earns £3 3s. or upwards a week plus expenses is erroneous and misleading. A good many medical men and most patients consider a minimum fee of £3 3s. exorbitant, and in very many cases a nurse is asked to reduce her fees.

However, admitting for the sake of argument that, provided her powers of endurance, mental and physical, were equal to the strain, a really well trained, well qualified, educated nurse might average ten months' work out of thirteen at an average fee of £13 13s. a month, her budget would work out approximately as follows: Fees earned, £136 10s., less rent for unfurnished room, £20; three months' board, light, and fire at £8 per month, £24; upkeep of uniform, trunks, books, instruments, etc., £10; telephone charges, £1; upkeep of furnishings, £5; making a total to be deducted of £60, so that the fees earned, less expenses, are £76 10s.

In order to earn rather less than £80 a year, plus all expenses, a private nurse must work very long hours, in many cases day and night, snatching sleep when she can between being called up. Her scanty off-duty, never more than two hours daily—often less—must be taken always at the patient's convenience.

By the nature of her duties and the conditions she lives under, a private nurse is debarred from participating in any social, intellectual, or political life whatever. She is called upon to adapt herself to varying degrees of domestic efficiency, and must be content to live a life almost entirely restricted to the four walls of a sick-room. She is constantly exposed to risk from infection, and when ill is entirely without income and must fall back on her scanty savings for support. The number of years she is capable of doing good work are few compared with the number of years she is incapable of working at such high pressure. If these facts are pondered over the scarcity of private nurses will be understood.

I am, Sir, yours faithfully,
"One Who Knows."

[Our correspondent's letter raises interesting points, and we hope that other private nurses and women workers in other spheres will state their experiences. "One Who Knows," however, confuses her figures. To her income it is only fair to add the cost of her board, lodging, and laundry for ten months (forty weeks)—certainly at present prices not less than £80. This brings her total earnings to £216 10s. How many members of the teaching profession, for instance, earn more? And how many women workers are there who, after necessary expenses are paid, have £76 10s. in hand for mufti, amusements, personal travelling, and saving, spread over a period of twelve weeks in each year?—Ed. T.H.]

